# Recent Advances in Localized Immunomodulation Technology: Application of NIR-PIT toward Clinical Control of the Local Immune System

**DOI:** 10.3390/pharmaceutics15020561

**Published:** 2023-02-07

**Authors:** Mizuki Yamada, Kohei Matsuoka, Mitsuo Sato, Kazuhide Sato

**Affiliations:** 1 Division of Host Defense Sciences, Department of Integrated Health Sciences, Nagoya University Graduate School of Medicine, Nagoya 461-8673, Japan; 2 B3 Unit Frontier, Advanced Analytical and Diagnostic Imaging Center (AADIC)/Medical Engineering Unit (MEU), Nagoya University Institute for Advanced Research, Nagoya 466-8550, Japan; 3 FOREST-Souhatsu, CREST, JST, Tokyo 102-0076, Japan; 4 Graduate School of Medicine, Nagoya University, Nagoya 466-8550, Japan

**Keywords:** near infrared photoimmunotherapy, local immunomodulation, regulatory T cells, immunotherapy

## Abstract

Current immunotherapies aim to modulate the balance among different immune cell populations, thereby controlling immune reactions. However, they often cause immune overactivation or over-suppression, which makes them difficult to control. Thus, it would be ideal to manipulate immune cells at a local site without disturbing homeostasis elsewhere in the body. Recent technological developments have enabled the selective targeting of cells and tissues in the body. Photo-targeted specific cell therapy has recently emerged among these. Near-infrared photoimmunotherapy (NIR-PIT) has surfaced as a new modality for cancer treatment, which combines antibodies and a photoabsorber, IR700DX. NIR-PIT is in testing as an international phase III clinical trial for locoregional recurrent head and neck squamous cell carcinoma (HNSCC) patients (LUZERA-301, NCT03769506), with a fast-track designation by the United States Food and Drug Administration (US-FDA). In Japan, NIR-PIT for patients with recurrent head and neck cancer was conditionally approved in 2020. Although NIR-PIT is commonly used for cancer therapy, it could also be exploited to locally eliminate certain immune cells with antibodies for a specific immune cell marker. This strategy can be utilized for anti-allergic therapy. Herein, we discuss the recent technological advances in local immunomodulation technology. We introduce immunomodulation technology with NIR-PIT and demonstrate an example of the knockdown of regulatory T cells (Tregs) to enhance local anti-tumor immune reactions.

## 1. Introduction

Immunomodulation can theoretically be used to target specific cells, but off-targets and side-effects can occur. Therefore, a practical method for local immune manipulation in the body is required. Genetic engineering has been used to control the immune system, such as by knockout (including conditional knockout) or implanting special engineered biomaterials or nanoparticles to modify the local immune system, which has been difficult to translate to a clinical setting, where ethical or clinical requirements are significant [1]. If localized immunomodulation could be used in humans, we could eradicate cancers without severe adverse events and apply it to the treatment of autoimmune diseases, allergies, and graft-versus-host disease in organ or tissue transplantation. It could also be further applied in immunology studies to clarify the mechanism of acquired autoimmune diseases, resulting in the emergence of new scientific fields [2]. In this review, we focus on recent developments in immunomodulation technology for the control of the immune system and to treat autoimmune disorders and inflammation.

### 1.1. Need for Methods of Local Immunomodulation

In clinical situations, immunotherapy is often performed systemically, causing treatment-limiting autoimmune or infectious events [3,4,5]. For example, immune checkpoint molecules can disrupt the balance of immune tolerance and lead to immune-related adverse events (irAEs) in normal organs, causing hypothyroidism, hypophysitis, pneumonitis, pancreatitis, and enterocolitis [6,7]. Anti-inflammatory agents cause immunosuppression in normal organs, which could lead to infectious pneumonia, hepatitis, and kidney injuries [3]. Thus, immunomodulation solely of target organs or tissues is highly desirable. A variety of approaches have been used to modulate immune reactions, but these have limited success. For instance, methods of organ-specific gene knockout, gene transfer with nanoparticles, and engineered biomaterials have been attempted and exploited [8,9]. Despite recent progress, it is difficult to translate these findings into clinical practice. Therefore, we need an innovative method that can be transferred to a clinical setting.

### 1.2. Recent Technologies for Local Immunomodulation

Regulation of the immune response or tolerance is difficult. As many players work in the immune system, complex biological reactions are generated. A variety of specific immune cells, such as B cells, T cells, dendritic cells (DCs), myeloid-derived suppressor cells (MDSCs), regulatory T cells (Tregs), and macrophages have been tweaked in earlier studies. In addition to immune cells, cytokines and enzymes have also been modified. Some methods have achieved great outcomes for the treatment of various disease models; however, they are often difficult to translate into the clinic because of their toxicity, biodistribution, and solubility [10,11,12,13].

Nanotechnology-based medicine has been widely developed in the past few years and offers the following advantages [14,15]: nano-drugs can combine diagnostic functions and therapy with theranostic agents; a targeting function can be added to improve accumulation; bioavailability, biodistribution, and clearance can be modified to improve therapeutic efficacy; and drug release can be controlled with a manageable time range, dose, and location [16]. By exploiting these nanotechnologies, various approaches to modulating local immune systems have been developed (Table 1). However, these technologies still present some obstacles for clinical use in humans. Another recent emerging technology that could be applied clinically is a phototechnology for ablation of specific cells inside the body, which could succeed in the spatiotemporal modulation of immune reactions [17].

### 1.3. Near-Infrared Photoimmunotherapy (NIR-PIT)

**Figure 1:**
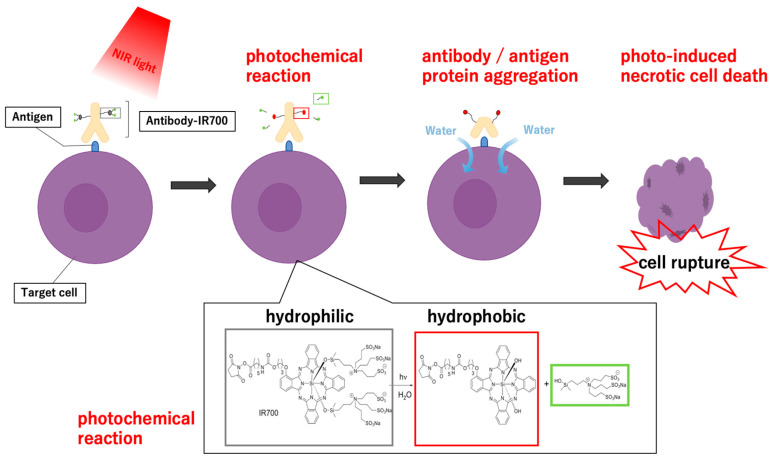
Schematic diagram for cellular cytotoxicity induced by near-infrared photoimmunotherapy (NIR-PIT). First, antibody-IR700 conjugates bind to molecules on the cancer cell membrane; when exposed to NIR light, a photochemical reaction occurs. These photochemical reactions can dramatically alter the physical characteristics of reactive molecules. In detail, the side-chain silanol groups of hydrophilic silicon phthalocyanine are released, changing IR-700 from hydrophilic to hydrophobic. Then, the added antibody proteins aggregate along with the antigens bound to the antibody’s gate, causing physical damage to the cell membrane. Finally, the water outside the cell flows into the cell to burst it, leading to necrotic cell death.

Photo-inducible immune modulation has recently emerged as a method for local immunomodulation. The concept of targeted light therapy was introduced around three decades ago [16]. Traditional photodynamic therapy (PDT) has been used as a hydrophobic photosensitizer; therefore, the pharmacokinetics of antibody-conjugated conventional PDT agents limit their ability to target selectively and their biodistribution on the target site [35,36]. As for NIR-PIT, since IR700 is a hydrophilic photoabsorber, the conjugates do not alter the antibodies’ biodistribution in the body, nor do they decrease the specific binding affinity on the target protein. Due to their hydrophilicity, they cannot pass through the lipid cell membrane; NIR-PIT excites the photochemical reaction on the membrane. Since these pharmacodynamics and pharmacokinetics are different from conventional photo-therapies, a “new” method of tumor therapy using NIR light was born, in which the cell death mechanism is unique (Figure 1) [17]. The wavelength used in NIR-PIT is 690 nm, which is a relatively longer wavelength able to reach deeper into the tissue than lower-wavelength light [37,38]. These antibody-photoabsorber (IR700) conjugates have similar intravenous pharmacokinetics to unconjugated antibodies, resulting in highly targeted tumor accumulation without off-target binding [39,40,41,42,43]. After binding to the targeted cells, the conjugates induce rapid, selective cytotoxicity upon exposure to NIR light [44,45,46,47,48,49,50]. Mixed culture models in vitro and in vivo have revealed no side effects on off-target cells or tumors [51,52]. This selective and rapid necrosis is unique to NIR-PIT in contrast to other anti-cancer therapies [53,54]. Recently, it has been clarified that the photochemical reaction induces an acute molecular change in the photoabsorber, converting it from a hydrophilic state to a hydrophobic one and resulting in damage to the cell membrane [55].

**Figure 2:**
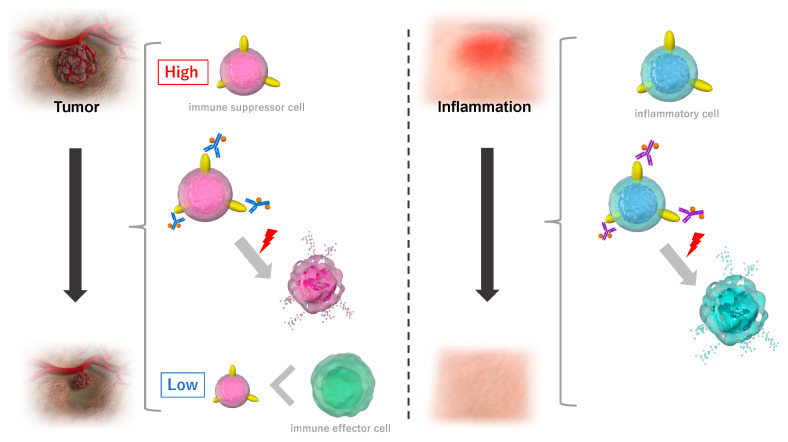
Applications of NIR-PIT as a technique that can target and locally eliminate specific immune cell subsets. Schematics of NIR-PIT as it activates local immune responses and induces anti-tumor responses by targeting immune suppressor cells in the tumor microenvironment (**left**), and targets inflammatory immune cells to reduce tissue damage (**right**).

This new therapeutic modality is now being tested in an international phase III clinical trial for locoregional recurrent head and neck squamous cell carcinoma (HNSCC) (LUZERA-301, NCT03769506). This trial is authorized with fast-track and SAKIGAKE by the United States Food and Drug Administration (US-FDA) and the Japanese Pharmaceuticals and Medical Devices Agency (PMDA), respectively [37,56]. With these priorities for approval, NIR-PIT was conditionally approved by the Japanese PMDA in 2020. Although NIR-PIT was originally developed to treat cancers, this method can be applied to the local knockdown of specific immune cell subsets. With this application, local immunomodulation can be achieved in humans [57]. NIR-PIT could augment local immune reactions by targeting specific immune suppressor cells and could reduce tissue damage by targeting inflammatory immune cells (Figure 2). 

### 1.4. Unique Necrotic Cell Death Mechanism in NIR-PIT

The therapeutic mechanism of NIR-PIT is very different compared to existing cancer therapies. Conventionally, oxidative stress is thought to be the main mechanism of photosensitizer-induced cell death. This pathway is thought to be cellular damage caused by oxidative stress agents such as free radicals and singlet oxygen (type I and type II) [58,59]. The energy difference between photoexcitation and photoemission of photosensitizers acts on mitochondria and other structures mainly inside the cells, leading to cell apoptosis [60]. This oxidative stress would affect not only target cells but also non-targeted adjacent cells.

In NIR-PIT, however, the chemical property of IR700 (hydrophilic) is rapidly changed to hydrophobic by the release of the silanol side chain under NIR light, and the hydrophobic IR700 aggregates in aqueous solution, resulting in the loss of IR700 fluorescence from the solution, which is considered to be related to a series of photochemical reactions. This hypothesis was proven using analytical chemistry and nano-in-liquid imaging [55].

After NIR light irradiation, free silanol groups were detected in both the supernatant of the IR700 solution and the antibody–IR700 conjugate solution (cetuximab-IR700: cet-IR700); furthermore, those in the antibody–IR700 conjugate solution were not derived from the antibody but from IR700.

SDS-PAGE analysis to examine the effects of the photochemical reaction on antibody and antigen proteins revealed that the photochemical reaction of IR700 caused protein denaturation in the form of aggregation of both the added antibody protein and the antigen–antibody complex. This confirms that the antibody–IR700–antigen complex aggregates upon photoirradiation.

Furthermore, imaging of a single antibody molecule by atomic force microscopy (AFM), which allows nanoimaging in liquids, also demonstrated that the cell membrane damage from NIR-PIT is due to the direct photochemical action of the antibody–IR700 conjugate.

In addition, IR700 fluorescence, which is reduced after NIR light irradiation, correlated with cytotoxicity in target cells in vitro and in target tumors in vivo. Thus, these results suggest that the IR700 axial ligand release response may result in severe physical changes within the antibody–IR700–antigen complex and cause cell membrane damage to target cells. This fluorescence change in IR700 is now being tested as a therapeutic biomarker in a clinical trial (NCT05182866). Collectively, it was found that NIR light dramatically changes the hydrophilicity of IR700 through its photo-induced axial ligand release reaction, which induces a change in the shape of the conjugate and its tendency to aggregate in aqueous solution in the body. When this photochemical reaction happens in the conjugates bound to cell membrane receptor antigens, they suddenly aggregate upon near-infrared irradiation and induce physical cell membrane damage via protein-denaturing cell membrane antigens, causing cell rupture, increased transmembrane water flow, and cell death. This mechanism can be proposed as a new light-induced cell death that does not depend on oxygen and is completely different from the oxidative stress proposed in conventional phototherapy, establishing a theory that NIR-PIT has advantages as a new anti-cancer modality that is different from previous cell-death concepts.

## 2. Light Irradiation Enabling Localized NIR-PIT

For effective NIR-PIT, the irradiation of NIR light onto the target tissue is a very important issue. The depth of penetration of NIR light into tissues is approximately 2~3 cm from the surface and it is significantly attenuated with distance. Moreover, light scattering via skin surface and lipid layers also decreases light energy. Therefore, theoretically, NIR-PIT is most suitable for the treatment of body-surface diseases such as those of the oral cavity and skin, and can be applied with conventional extracorporeal devices with a frontal diffuser [61]. In frontal diffusers used for surface treatment, the laser beam is directed from the light source to the distal end to obtain a uniform irradiation spot at the tip end.

Accurate irradiation with NIR light and its evaluation are required in order to only produce the NIR-PIT effect on the treatment target without adversely affecting normal tissues, even in deep areas. Here, we summarize information on light irradiation enabling localized NIR-PIT, focusing on the development of various devices as examples of the application of NIR-PIT to deep-area treatment of tumors.

The light sources which are now being used for NIR-PIT are LEDs and lasers. Although laser light has a higher-coherency, more-stable monochromatic wavelength, it has a narrower beam and costs more compared to LEDs. On the other hand, LEDs can irradiate wider-spread areas and are cheaper than lasers; however, they have wider bandwidth and more unstable and variable wavelengths, amplitude, and phase, which is a disadvantage compared to lasers [62]. Since with the same light energy the effect of NIR-PIT by lasers is greater than with LEDs and we can exploit lasers for clinical use via many catheters, we prefer lasers as a light source for NIR-PIT [45].

In tissue-irradiation diffusers used for in-tissue treatment, the laser beam is guided from the light source to the distal end and uniformly dispersed from the side of the optical fiber at the tip. A cylindrical irradiation tip is employed for this, and the laser beam guided by the optical fiber is uniformly irradiated from the end of the optical fiber in the circumferential direction of the optical fiber, exposing a wider area than the front illumination tip [63]. Additionally, needle catheters with a sharp, closed-tip laser light-transmitting resin needle and a stainless steel obturator can be used to introduce a tissue-irradiation diffuser into the tissue in intra-tissue therapy. Fiber-optic optical diffusers placed in needle catheters can be inserted into deep tumors, and this technique has been used in clinical practice [64].

Another option is to administer NIR light endoscopically. The use of an endoscope and fiber-optic diffuser is a promising tool for delivering NIR light in tumors within or adjacent to hollow organs in a minimally invasive and safe manner. In gastrointestinal diseases, the efficacy of endoscopic NIR-PIT using trastuzumab–IR700DX (tra-IR700) and a cylindrical light diffuser was demonstrated in a mouse model of human epidermal growth factor 2 positive (HER2+) gastric cancer with peritoneal disseminations [65,66]. This suggests that NIR-PIT can be applied to treating tumors that cannot be resected endoscopically, either for technical reasons such as inability to reach anatomical areas or because the tumor invades deep muscular layers and cannot be completely removed. In head and neck disease, the utility of endoscopic NIR-PIT was demonstrated in an orthotopic head and neck cancer (HNC) model using the CD44-expressing MOC2-luc cell line, along with the utility of real-time quantification of fluorescent signals by endoscopy [67]. Furthermore, in respiratory diseases, optical fibers are utilized through bronchoscopes to deliver NIR light to key areas, and recent bronchoscopic technology can provide NIR light to almost any intrapulmonary lesion. This bronchoscopic technique can be navigated with a 3D navigation system from prior CT images to accurately guide the fiber to the tumor in the lung. These techniques minimize damage to normal lung fields [56,68].

Light-irradiation devices that enable local NIR-PIT are increasingly being developed, ranging from a new catheter with light-emitting diode (LED) to an endovascular therapy-based light illumination technology (ET-BLIT) to an implantable wireless-powered LED. These new technologies give us choices to fit the patients’ disease situations. 

As mentioned above, while the endoscopic approach has the potential to be effective in irradiating NIR light in various sites, delivery of laser NIR with a light diffuser into deep lesions in retroperitoneal organs such as the bile duct, pancreas, urinary tract, and spleen is difficult when using an endoscope because implanting a light diffuser in these organs is highly invasive and it is difficult to repeatedly irradiate tissues with a continuous indwelling. Furthermore, when fiber-optic diffusers are used, there is a risk of kinking during deep insertion. However, a new type of catheter with LEDs mounted on flexible printed circuit boards is flexible and does not kink, allowing deep insertion and irradiation using an endoscope or guidewire. Therefore, a new catheter with LED was developed as a new light-illuminated flexible catheter that can be safely implanted long-term using an assist device and overcomes the limitations of conventional external irradiation devices and endoscopic diffusers. It also features a temperature sensor to avoid thermal injury. Using this catheter, tumor growth of cholangiocarcinoma xenografts in treated mice was significantly inhibited, proving the effectiveness of this novel device [69]. Other developments include ET-BLIT, a system that delivers light deep into the body. The ET-BLIT catheter system consists of two distinctive devices: a partially transparent catheter tip with a transparent distal end attached to a thermocouple head with an outer diameter of approximately 1 to 5 mm able to fit multiple vessels and a light diffuser having a luminescent function on the distal portion. The basic procedure with this system is comparable to existing endovascular therapies, making it a promising potential way to enable to bring light anywhere inside the body for clinical use. Tests in animal models have proven the effectiveness of this system, with NIR light penetrating the walls of blood vessels and reaching the liver and kidneys without causing temperature increases, vascular damage, or changes in blood composition [70]. The development of endoscopes and catheters for localized NIR-PIT of deep lesions is remarkable; however, it is a little bit invasive given the need for repeated treatments. In fact, repeated treatment or repeated light exposure with a single dose of antibody–photoabsorber conjugates can produce better therapeutic effects than a single treatment or single light exposure [40,50,71]. Implantable wireless powered LEDs were developed to limit the invasive nature of repetitive treatments in deep tissues while at the same time maintaining efficacy. Here, to avoid the potentially harmful implantation of batteries, power to the embedded LED was ensured by electromagnetic induction from an external transmitter coil coupled to the LED to an embedded receiver coil. The LED system was shown to kill tumor cells in vitro and inhibit tumor growth in implanted tumor-containing mice. Thus, it was found that implantable wireless-powered LEDs may be a possible solution for treating tumors in deep areas of the human body [70].

Local NIR-PIT without specialized devices includes NIR-light irradiation during surgical procedures. The introduction of intraoperative NIR-light irradiation eliminated microscopic disease of the tumor bed and prevented local and metastatic recurrence [72].

Thus, along with the use of a variety of advanced devices and in combination with other treatment modalities, it would be possible to perform local NIR-PIT appropriate for each of the broad areas of the body. Of note, the developed light devices are also utilized with other wavelengths for other photo-based therapies.

## 3. Demonstration of NIR-PIT for Local Immunomodulation: Targeting Tregs at the Tumor Site

Here, we discuss an example of a local immune augmentation against tumors with localized knockdown of CD4^+^CD25^+^Foxp3^+^ Tregs [73]. NIR-PIT using an antibody–photoabsorber conjugate that targets CD25 selectively depletes the CD4^+^CD25^+^Foxp3^+^ Tregs infiltrating the tumor microenvironment and activates CD8^+^ T cells and natural killer (NK) cells, resulting in local anti-tumor immunity. In this treatment, Tregs were selectively killed, but non-activated CD8^+^ T cells and NK cells infiltrating the tumor remained unaffected. Thus, local CD25-targeting NIR-PIT can activate effector cells without systemically eliminating the suppressor cells. These findings contrast with previous reports of systemic administration of anti-CD25 antibodies or IL-2-toxin conjugates to deplete systemic Tregs, which both depleted Tregs and activated effector cells that express CD25.

In the future, the combination of local CD25-targeted NIR-PIT with other immunomodulatory therapies, such as immune checkpoint inhibitors [74] or cancer-specific therapies, could be an effective therapeutic option. We therefore list some possible target molecules (CD25, CD103, CD73, CCR4, CTLA-4, GITR, and Gr1) for NIR-PIT that induce knockdown of immune suppressor cells, especially Tregs, in the tumor microenvironment in order to activate anti-tumor responses. Furthermore, we list some possible target molecules for the use of NIR-PIT as a localized immunomodulation therapy for non-tumor diseases such as allergies, inflammation, and autoimmune disorders.

### 3.1. CD25

CD25 receptors are expressed on the surface of Tregs, which is a key factor associated with immunosuppression of effector T and NK cells at the tumor site. F(ab’)2 fragments were purified to avoid Fc-mediated antibody-dependent cellular cytotoxicity (ADCC) and complement-dependent cytotoxicity (CDC) in vivo. Anti-CD25-F(ab’)2-based NIR-PIT was more effective in reducing tumor growth than anti-CD25-IgG-based NIR-PIT. Absence of the Fc portion of the APC leads to faster clearance and therefore promotes a superior activated T cell response in tumors [75].

### 3.2. CD103

CD103 is also known as αE integrin, which mediates lymphocyte retention in epithelial tissues. It was reported to be expressed at high levels in tumor-infiltrating Foxp3^+^ Tregs in several types of murine cancers. One of the hallmarks of TGF-β-secreting tumor-infiltrating Tregs is the expression of CD103, which is an interesting and important target for selectively reducing tumor-infiltrating Tregs [76].

### 3.3. CD73

Accumulated extracellular adenosine may cause the loss of cytotoxic potential of CD8+ T cells and NK cells in TME, and the major extracellular source of adenosine is CD73 [77]. CD73 is expressed at high levels on both tumor cells and on Tregs, MDSCs, and TAM.M2, but not on cytolytic T lymphocytes, NK cells, or DCs. NIR-PIT with anti-CD73 antibody simultaneously eliminates tumor cells and major immunosuppressive cells [78].

### 3.4. CCR4

CCR4 is predominantly expressed by effector Treg cells and not by naive Treg cells and Th2 cells, which do not contribute significantly to tumor-immunity regulation in humans. Cell-depleting anti-CCR4 mAb therapy is instrumental for evoking and enhancing tumor immunity in humans via selectively removing effector-type FOXP3^+^ Treg cells [79].

### 3.5. CTLA-4

CTLA-4 (cytotoxic T-lymphocyte-associated protein 4) is a very potent co-suppressor molecule that is constitutively expressed in Tregs and post-activation Tconv cells. CTLA-4 is transiently expressed in normal T cells upon activation.

The anti-CTLA-4 antibody may kill tumor-specific effector Treg cells or attenuate their suppressive activity [80]. Anti-CTLA-4 antibodies may eliminate tumor-specific effector Treg cells or deplete their inhibitory activity.

### 3.6. GITR

Treg cells constitutively express high levels of glucocorticoid-induced tumor necrosis factor receptor (GITR) compared with conventional T cells. The anti-GITR antibody in tumor-bearing mice can break immunological self-tolerance and evoke a potent anti-tumor immune response with an increase in IFN-γ-producing CD8+ and CD4+ T cells [80,81].

### 3.7. Gr1

MDSCs play an active role in the immune surveillance escape of cancer cells. In mice, MDSCs are broadly characterized as CD11b^+^Gr1^+^, with two major cell subsets, granulocytic MDSCs (G-MDSCs, CD11b^+^Ly6C^low^Ly6G^+^) and monocytic MDSCs (M-MDSCs, CD11b^+^Ly6C^high^Ly6G^−^). NIR-PIT can be used to eliminate splenic MDSCs, identifying its potential to eliminate MDSCs in tumors to reduce immune suppression [82].

### 3.8. IL-5Rα(CD125)

The IL-5 receptor is a heterodimer of the IL-5Rα chain and the common β chain shared by the IL-3 and GM-CSF (granulocyte-macrophage colony-stimulating factor) receptors. The increasing expression of IL-5Rα in CD34+ cells favors eosinophilopoiesis and may thus contribute to the subsequent development of blood and tissue eosinophilia, a hallmark of allergic inflammation [83].

Benralizumab, a humanized anti-IL-5 receptor alpha monoclonal antibody, targets the IL-5 receptor alpha (IL-5Rα), directly inducing cell cytotoxicity and depleting eosinophils, their progenitor cells in blood, bone marrow, and airway tissue, and other IL-5 receptor-bearing cells such as basophils, improving symptoms in patients with severe eosinophilic asthma [84].

### 3.9. IL-4 Receptors

The IL-4 receptor (IL-4R) has two types, type I and type II, and IL-4Rα is the common subunit. Dupilumab, which targets IL-4Rα, suppresses IL-4/IL-13 signaling that induces skin barrier dysfunction and cytokine production. Inhibition of IL-4/IL-13 signaling by dupilumab-mediated IL-4 receptor alpha blockade significantly improved atopic/allergic disease activity. Moreover, it suppressed systemic type II inflammatory indices and epidermal abnormalities [85,86].

### 3.10. RANKL

In an inflammatory environment, such as rheumatoid arthritis, immune-suppressing CD25loFoxp3+ CD4+ T cells lose Foxp3 expression and convert to immune-promoting exFoxp3 Th17 cells, which express IL-17 and promote immunity. exFoxp3Th17 cells express high levels of membrane RANKL on the cell surface [87].

## 4. Conclusions

In this review, we discussed the challenges of local immunomodulation using recent technologies. Although some methods have achieved great outcomes for the treatment of various disease models, they are often difficult to translate into the clinic because of their toxicity, biodistribution, and solubility. Thus, we suggest the possibility of using NIR-PIT, which could enhance local anti-tumor immune reactions by depletion of Tregs, for local immunomodulation. Possible targets for local immunomodulation by NIR-PIT depend on the development of the target moieties. In future, NIR-PIT using these targets would enhance its usage for not only anti-tumor immune reactions but also allergies, autoimmune diseases, and organ transplants.

## Figures and Tables

**Table 1:** Recent technologies for local immunomodulation.

Methods	Examples	Characteristics	Ref.
Methods using synthetic biomaterials	PLG scaffolds	**<PLAGA+Treg>** Co-localizing immunomodulatory cells with islets to affect the immune system on a local and systemic level.	[18]
		**<PLGA+TGF-β>** Localized TGF-β1 delivery modulates the immune response to biomaterial implants and enhance cell function in cell-based therapies.	[19]
	PEG biomaterials	**<Peptide-functionalized PEG hydrogels>** (TNFα-antagonizing PEG- WP9QY hydrogels/MCP-1-antagonizing PEG-WKNFQTI hydrogels) PEG hydrogels presenting peptides which sequester the pro-inflammatory cytokine TNFα and chemokine MCP-1 reduce the host’s innate immune response to transplanted cells by decreasing the recruitment and activation of immune cells.	[20,21]
		**<Drug-PEG-coating>** (Rapa-PEG-coating around alginate microspheres) PEG biomaterials are used to locally deliver drugs that suppress the immune response.	[22]
		**<SA-FasL microgels>** SA-FasL locally immunomodulates (increase ratio of Treg to CD4+ and CD8+ Teff cells) in allograft.	[2]
Methods using nanoparticles	pTrap LCP	**<pDNAs encoding PD-L1 and CXCL12 traps into the LCP vector>** Greatly increased the concentrations of immunotherapeutic agents in local tissue, allowing the therapy to inhibit the accumulation of immune-suppressive cells.	[9]
	Nanosuspension	**<Hyaluronic Acid—Dexamethasone Nanoparticles>** Local delivery directly to the lung in the form of liquid aerosol administered into the pipe of the ventilator.	[23]
	Protein nanogels (NGs)	**<Cell surface-conjugated NGs>** The use of cell surface-conjugated protein nanogels (NGs) responsive to T cell receptor (TCR) activation as a local source of adjuvant could expand T cells in tumors and increase cytokine administration without toxicity, greatly improving therapeutic efficacy.	[24]
Methods of genetic engineering	Gene immunotherapies	**<pCXCL12 trap / pPD-L1 trap>** Increases the activation of cancer-specific CD8þ T-cells by gene immunotherapies.	[9]
	TRECK	**<Mas-TRECK and Bas-TRECK mice>** Specifically destroying target cells (mast cells/basophils) at an arbitrary time by creating transgenic mice in which a human-derived diphtheria toxin receptor (DTR) gene is introduced into the mouse downstream of a cell or organ-specific promoter.	[25]
(and photothermal control)	Engineered CAR T cells	**<Bispecific T cell engager bearing an NKG2D receptor>** The activity of intratumoral CAR T cells can be controlled photothermally via synthetic gene switches.	[26]
Targeting the extracellular matrix	aTM	An artificial T cell-stimulating matrix (aTM), composed of a hyaluronic-based hydrogel with tunable stiffness; further promotes T cell expansion.	[27]
	T cells expressing CAR against FAP	Adoptive transference of **T cells** expressing a chimeric antigen receptor (CAR) against FAP; endogenous CF target specifically targets pathologic cardiac fibrosis.	[28]
	Engineered ECM-binding checkpoint blockade Abs	**<PlGF-2123–144–anti-CTLA4 / PlGF-2123–144–anti–PD-L1 Abs>** These engineered ECM-binding checkpoint blockade Abs locally increase tumor-infiltrating activated CD8+and CD4+T cells.	[29]
	Engineered cytokine–ECM fusion systems	**<CBD-IL-2 and IL-12>** A collagen-binding domain fused to IL-12 (CBD-IL-12) by direct injection into the tumor mass/intravenous injection prolongs and localizes therapeutic antitumor activity within the tumor microenvironment.	[30,31]
Method using compounds	Carvone (fragrance compound)	Some compounds have different immunomodulatory properties (differences in activation and suppression/ CD3+Tcell, cytokine variation) in the hippocampus among individuals with genetic variability, mutations, and polymorphisms.	[32]
Method using antimicrobial peptide	LNIT	**<Local nasal immunotherapy (LNIT) with FIP-fve and DN-Tp>** LNIT with FIP-fve and DN-Tp had an anti-inflammatory effect on mite-induced airway inflammations and possesses potential as an immunomodulatory therapy agent for allergic airway diseases.	[33,34]

## Data Availability

Not applicable.
